# Supplementary material for: In-Hospital Mortality in Non-COVID-19-Related Diseases before and during the Pandemic: A Regional Retrospective Study
Source: Int J Environ Res Public Health. 2021 Oct 16;18(20):10886. doi: 10.3390/ijerph182010886 (PMC8535491; doi:10.3390/ijerph182010886)
Supplement: Supplementary file 1 [file ijerph-18-10886-s001.zip › ijerph-1393839-supplementary.pdf]

Table S1. Observed deaths, in-hospital mortality standard rates and standardized mortality rate ratios by fortnightly period of discharge.

Fortnight	2019		2020		SRR	95%CI
	Obs. Deaths	IMR±SE	Obs. Deaths	IMR±SE		
January 1 – January 15	681	42.28±1.63	648	42.79±1.69	1.012	0.908-1.128
January 16 – January 31	767	34.54±1.25	685	31.58±1.21	0.914	0.825-1.014
February 1 – February 15	690	33.25±1.27	625	31.82±1.28	0.957	0.859-1.066
February 16 – February 28	538	31.7±1.37	523	28.5±1.25	0.899	0.797-1.014
March 1 – March 15	640	31.72±1.26	624	36.94±1.48	1.164	1.043-1.3
March 16 – March 31	667	32.91±1.28	555	56.75±2.45	1.724	1.539-1.932
April 1 – April 15	497	25.21±1.13	457	57.76±2.73	2.291	2.016-2.603
April 16 – April 30	544	31.34±1.35	447	48.78±2.33	1.557	1.373-1.765
May 1 – May 15	499	27.22±1.22	425	43.68±2.13	1.605	1.409-1.827
May 16 – May 31	536	25.5±1.1	442	35.39±1.69	1.388	1.223-1.574
June 1 – June 15	547	28.11±1.2	414	31.55±1.56	1.123	0.988-1.276
June 16 – June 30	502	27.55±1.23	436	28.77±1.38	1.045	0.918-1.188
July 1 – July 15	507	27.71±1.23	426	27.57±1.34	0.995	0.874-1.132
July 16 – July 31	529	27.18±1.18	492	29.48±1.33	1.085	0.959-1.227
August 1 – August 15	512	32.96±1.46	461	34.6±1.62	1.050	0.925-1.191
August 16 – August 31	528	33.96±1.49	508	39.67±1.77	1.168	1.034-1.32
September 1 – September 15	479	30.1±1.38	479	34.39±1.58	1.142	1.006-1.297
September 16 – September 31	516	28.61±1.26	459	30.74±1.44	1.074	0.947-1.219
October 1 – October 15	464	25.47±1.18	406	27.04±1.35	1.062	0.929-1.214
October 16 – October 31	486	24±1.09	503	32.4±1.46	1.350	1.191-1.53
November 1 – November 15	509	29.15±1.29	489	41.1±1.87	1.410	1.245-1.597
November 16 – November 30	507	27.21±1.21	516	44.1±1.96	1.621	1.432-1.833
December 1 – December 15	503	28.55±1.27	523	46.55±2.05	1.630	1.442-1.844
December 16 – December 31	619	35.45±1.43	582	48.72±2.03	1.375	1.227-1.54

IMR: in-hospital mortality standard rates; SE: standard error; SRR: standardized mortality rate ratio; CI: confidence interval.
